# Supplementary material for: Are carbon emissions trading and green financial instruments synergistic? -Comprehensive quantitative research based on content analysis
Source: PLoS One. 2024 Mar 7;19(3):e0298601. doi: 10.1371/journal.pone.0298601 (PMC10919601; doi:10.1371/journal.pone.0298601)
Supplement: S1 Data — (ZIP) [file pone.0298601.s001.zip › Data/地级市Z_F工作报告.zip!/║╙▒▒/║¬╡a╩╨/└┤╫╘╣1⁄2╓┌║┼í╛┬φ┐╦╩2╛▌═°í┐_2019.docx]

各位代表：

现在，我代表邯郸市人民政府，向大会作政府工作报告，请予审议，并请各位市政协委员和列席人员提出意见。

一、2018年工作回顾

刚刚过去的2018年，是全面贯彻落实党的十九大精神开局之年。我们坚持以习近平新时代中国特色社会主义思想为指导，认真贯彻党中央、国务院重大决策，全面落实省委、省政府部署要求，在市委的坚强领导下，在市人大、市政协的监督支持下，在全市广大干部群众的共同努力下，围绕新时代全面建设富强邯郸、美丽邯郸的战略目标，坚持稳中求进工作总基调，按照“三个三”总体思路，攻坚克难，实干实政，较好完成了市十五届人大三次会议确定的目标任务。

（一）全力以赴抓项目、稳增长，综合经济实力稳步提升。始终把项目建设作为发展的“生命线”，坚持“三集中”机制不动摇，用好“四色”管理法，争列省重点项目48个，数量连续4年居全省第一，实施市重点项目310个，完成投资1140亿元，占年计划的142%，发展后劲进一步增强。着力稳就业、稳金融、稳外贸、稳外资、稳投资、稳预期，经济运行在下行压力加大中实现稳中有进。全市生产总值、固定资产投资均增长6.6%，规模以上工业增加值增长4.5%；全部财政收入突破400亿元大关，达到438.1亿元，增长19.1%，税收占比提高2.8个百分点；一般公共预算收入243.4亿元，增长10.6%，税收占比提高2.5个百分点；新增贷款274.6亿元，增长7.3%；社会消费品零售总额完成1686.8亿元，增长9.3%；出口总值完成99亿元，增长8.2%。预计，规模以上工业企业实现利润288.4亿元，增长33.7%；城镇、农村居民人均可支配收入分别达31075元、14268元，分别增长8%和8.5%。

（二）坚定不移调结构、转方式，供给侧结构性改革迈出新步伐。坚持破立结合、消长并举，制定实施去产能、工业转型升级、战略性新兴产业发展、质量强市等系列三年行动计划，邀请国务院发展研究中心进行产业升级路径研究，进一步强化了高质量发展的顶层设计。压减退出炼铁产能151万吨、炼钢产能34.6万吨、煤炭产能115万吨、焦炭产能180万吨、火电装机规模26.8万千瓦，提前超额完成省定任务。技改投资增长15.1%，列入省级智能制造试点示范项目20个，六大高耗能行业增加值占规模以上工业比重下降1.6个百分点，高新技术产业增加值增长17.5%。服务业增加值增长10%，对经济增长的贡献率达71.5%。华耀城、美乐城、浙江商贸城等现代服务业项目投入运营，邯郸人力资源市场·产业园开园。成功举办第十四届国际太极拳运动大会暨第三届市旅发大会、首届绿色发展论坛，全省全域旅游现场会在涉县召开，馆陶粮画小镇通过4A级景区景观质量评审，全市接待国内外游客6994.1万人次、实现旅游业总收入799.2亿元，分别增长19.4%和25.2%。三次产业比重由2017年的9.1:47.9:43，调整为9.1:45.1:45.8，产业结构由“二三一”转变为“三二一”。实施质量强市战略，荣获中国驰名商标4件、地理标志商标4件、河北知名品牌55项，峰峰矿区国家煤化工产品质量监督检验中心通过认定。

（三）坚持不懈治污染、减排放，生态环境质量持续改善。全面打响大气、水、土壤污染防治攻坚战，制定大气污染综合治理“1+29”方案、水污染防治“1+12”方案，扎实做好中央环保督察“回头看”、生态环境部强化督查等反馈问题整改，环境治理取得积极成效。突出抓好燃煤污染治理，完成清洁取暖工程29.36万户。累计关停整治“散乱污”企业30229家。在全国率先开展钢铁焦化企业超低排放改造，全面推进无组织排放治理，开展企业“绿色评估”，统筹实施重点行业二三季度差别化管控和秋冬季错峰生产。预计全社会煤炭消费量比上年削减600万吨左右，单位生产总值能耗下降7%左右。开展柴油车辆尾气治理，严格油品质量管控，对1240台国三排放标准柴油车和1224台未达到国三排放标准的非道路移动机械加装DPF颗粒物捕集系统，有铁路专用线的11家重点企业铁路运输平均占比达73%。强力推进“六个三”扬尘综合治理、“一区三边”集中整治，拆违755万平方米，在入市口建设6个综合服务区，在国省干线实施“三机一体”作业，露天焚烧红外视频监控系统、热点网格监管平台、乡镇环保所实现全覆盖。PM2.5平均浓度为69微克/立方米，下降19.8%，改善率居全省第二，超额完成省定目标，打赢了大气污染防治翻身仗，空气质量为6年来最好。全面落实河（湖）长制，国省考核断面总体达标率100%，县级以上城市集中饮用水水源达标率稳定在100%，全面完成集中式水源地违规项目治理。深化地下水超采综合治理，新增地下水压采能力1.34亿立方米。我市列入省江水利用改革试点市，全年利用江水3.29亿立方米。严守耕地红线，高压整治违法占地，清理整治违规“大棚房”12个、323座棚室，28处责任主体灭失矿山迹地修复绿化、中央环保督察“回头看”涉及17处矿山整改提升任务全部完成。持续推进“绿美邯郸”攻坚，高标准规划建设170平方公里的西部生态屏障，全市完成营造林82.5万亩，森林覆盖率达32.2%。我市成功争列中央财政支持北方地区冬季清洁取暖试点城市、全国建筑垃圾治理试点城市、全国城市黑臭水体治理示范城市、全国绿色货运配送示范工程创建城市、省优先发展公共交通示范城市创建单位，荣获国家资源循环利用基地称号。

（四）毫不动摇促改革、扩开放，经济发展活力明显增强。市级机构改革顺利完成，县级机构改革有序推进。深化“放管服”改革，全面推行“一趟清”“不见面”改革，实现“55证合一”，新设市场主体15.5万户，总量达72.7万户、增长14.5%，全国开展相对集中行政许可权改革试点调研座谈会在我市召开，被确定为全国跨部门“双随机、一公开”联合监管试点市。我市深化商事制度改革成效显著，馆陶县公立医院综合改革成效明显，受到国务院办公厅通报表扬。国企、金融、财税、投融资等领域改革稳步推进，全市国企“三供一业”分离移交和12家国企破产改制全部完成，成功争列石交所可转债试点，鸡泽农商行和3家村镇银行开业，引进金融机构5家，邯郸银行在邢台、秦皇岛设立分行。先后举办邯郸苏州招商引智恳谈会、中韩经济合作交流会、京津冀协同发展邯郸专场推介会、中关村股权投资协会“会长会”走进邯郸、浙商走进河北（邯郸）合作交流大会等重大招商活动，共签约项目504个，其中总投资超100亿元项目9个、超50亿元项目31个。积极推动争计划、争项目、争资金和对接央企活动，签约央企合作项目16个，与河北港口、新兴际华等省部属企业合作进一步深化。在韩国、美国、加拿大设立3家境外招商代理，市对外贸易综合服务园区成立，邯郸中欧班列成功开行，搭建了对外开放新桥梁。预计省级以上开发区主营业务收入、税收分别增长30%和38%，马头经济开发区获批省级高新技术产业开发区。创新驱动发展战略深入实施，北京大学邯郸创新研究院设立大数据与人工智能实验室、新能源材料与技术中心、人文社科发展研究中心、智能装备研究中心及产业基地，我市与中科院生态环境研究中心、北京市科学技术研究院、吉林大学、省科技厅签订战略合作协议。制定实施科技创新三年行动计划，打造综合创新生态体系，预计全市研发经费支出占生产总值比重达到1.15%，新增高新技术企业106家、科技型中小企业1489家，新增省级重点实验室、工程研究中心、企业技术中心等创新平台28个，邯郸学院、市第一医院、汉光重工公司设立博士后科研工作站，14项科研成果获得省科学技术奖，院士工作站达30家，成安、肥乡等5个县（区）列入省创新型试点县，省第一届产学研大会在我市召开。全市技术合同交易额139亿元，增长5倍，总量和增速均居全省第一。创新创业日趋活跃，新增省级科技企业孵化器6家、众创空间8家。研究制定支持民营经济发展12条政策措施，帮助企业解决1172个实际问题，提供103.1亿元“过桥”资金，降低融资成本13亿元。10家企业入选全国民营企业500强，数量占全省的42%，11家企业荣获省优秀民营企业称号。预计民营经济增加值完成2266亿元，增长6.6%。

（五）持之以恒强基础、补短板，城乡一体化进程加快。建立东区建设封闭运行机制，配套基础设施及中央商务区建设全面提速，市客运中心、市科技中心、邯山区一中新校区等竣工投用，市体育中心、市职工活动中心等开工建设，毛遂大街、荀子大街等道路建成通车。完善提升老城区，打通13条“断头路”，33个城中村改造启动实施，424个老旧小区完成改造，378个房地产开发遗留问题项目全部出列。邯郸植物园、紫山公园、新动物园开园，邯郸火车站新站房投用，太行山高速公路通车。我市荣获“2018中欧绿色智慧城市”先行奖，连续六届荣列“中国城市信息化50强”。电力、邮政、通信基础设施建设力度加大。深化城乡观摩拉练机制，深入推进县城建设三年攻坚行动，县城新区框架全面拉开，实施城建重点项目323个，完成投资342.1亿元，县城面貌日新月异。预计全市常住人口城镇化率达到56.7%左右。乡村振兴战略深入实施，粮食总产105.6亿斤，省、市现代农业园区数量分别达21个、42个，省级示范农业产业化联合体达22家，预计农业产业化经营率达69%、同比提高0.7个百分点，争列省级特色小镇7个，中国农大在曲周县设立农业绿色发展示范区，鸡泽县、邱县、魏县列入全国农村一二三产业融合发展先导区创建单位，全国农业产业化联合体现场观摩推进会在我市召开。农村承包地确权登记颁证基本完成，集体产权资产清查核实实现全覆盖，土地流转率达33.53%，比上年提高4.53个百分点，发展规模经营主体9317家、股份合作组织3034家、市级以上示范家庭农场430家、农村电商服务站2770家，有效激发了农村发展活力。“四好农村路”建设成效显著，新建改建农村道路1268.5公里，涉县荣获全国示范县，省级示范县达10个，全省现场会在我市召开。实施农村饮水安全巩固提升工程，解决了17.5万人饮水安全问题，实现农村垃圾环卫保洁公司化服务全覆盖，垃圾处理城乡一体化覆盖75%以上的乡镇，改造农村厕所15万座，农村人居环境持续改善。推进“强乡富村”工程，全市集体收入空白村占比由2014年的70%下降到18.1%。

（六）扎扎实实惠民生、防风险，社会发展大局和谐稳定。

全力推进脱贫攻坚，深化“一帮一”结对帮扶，推广“扶贫微工厂”、光伏扶贫等模式，全市投入财政扶贫资金7.4亿元，5.1万人稳定脱贫，魏县、广平、馆陶、鸡泽、肥乡5个县（区）退出贫困县序列，大名县脱贫“摘帽”完成省级验收。我市“扶贫微工厂”做法被《中国扶贫》杂志列为“脱贫攻坚100计”第1计向全国推介，精准防贫工作被中央电视台《焦点访谈》及新华社、国务院扶贫办等多家媒体和机关专刊登载。财政民生支出427亿元，增长14.1%，20项民心工程全部完成。城市棚户区改造开工22429套、基本建成10453套，农村危房改造竣工2239户。新增城镇就业13.6万人，下岗失业人员再就业3.88万人。退休人员养老金待遇人均提高5.5%，城乡居民基础养老金标准每人每月增加18元，城乡居民基本医疗保险人均年财政补助标准提高到490元，农村低保平均标准提高12%，新建10家社区和居家养老服务中心。全市新建、改扩建公办幼儿园60所、中小学校舍13万平方米，学前三年毛入园率达90%。健康邯郸建设取得新进展，荣获全国基层中医药工作先进市、省卫生城市，连续9次荣获全国无偿献血先进市，连续4年荣获省医改工作先进市。文体事业蓬勃发展，新增中国历史文化名村5个、传统村落13个、省级历史文化街区2处、省级文物保护单位7处，我市运动员在省第十五届运动会上团体总分名列全省第三，成功申办省第十六届运动会。建立了“1+5+1”金融风险防控体系，严厉打击违法违规金融活动，设立政府举债融资正负面清单，完成市县存量政府债务置换工作。扎实开展扫黑除恶专项斗争，打掉5个涉黑犯罪组织、39个恶势力犯罪集团、28个恶势力团伙，刑事案件发案率下降10.4%。高度重视信访稳定，安全生产和食品药品安全形势总体平稳，依法关闭60座铁矿、23座采石场、10座尾矿库，生产安全事故起数和死亡人数分别下降15.1%和25%。驻邯部队全面停止有偿服务工作圆满完成，退役军人服务管理工作扎实有效，“两站两中心”服务管理机构在全省率先实现全覆盖，国防动员和双拥共建深入开展。圆满完成村、社区“两委”换届。民族宗教、新闻出版、广播电视、外事侨务、史志档案、人民防空、防灾减灾、援藏援疆、妇女儿童、老龄、残疾人等工作都取得了新成绩。

（七）驰而不息转作风、优服务，政府自身建设不断加强。深入学习贯彻习近平新时代中国特色社会主义思想和党的十九大精神，认真做好中央、省委巡视反馈意见整改，严格落实中央八项规定及其实施细则精神，深入推进“一问责八清理”专项行动整改“回头看”、纠正“四风”和作风纪律专项整治，扎实开展“新时代新担当新作为”主题活动，推出一批惠民便民新举措，机关作风明显改善。推进法治政府建设，认真贯彻落实《宪法》，制定完善《市政府党组工作规则》和《市政府工作规则》，组建了政府智库，自觉接受市人大及其常委会法律监督和工作监督、市政协民主监督和社会舆论监督，科学民主依法决策水平进一步提高。办理人大代表建议426件、政协提案612件，办结率100%。全力支持纪检监察机关工作，切实加强审计监督，反腐倡廉力度不断加大。扎实推进“双创双服”活动，合力扶工助企，服务型政府建设取得新成效。

各位代表！过去的一年，我们激情奋斗、砥砺前行，顶住了压力，经受了考验，取得了来之不易的成绩。这是习近平新时代中国特色社会主义思想科学指引的结果，是省委、省政府和市委坚强领导的结果，是市人大、市政协和社会各界鼎力支持的结果，是全市广大干部群众撸起袖子、挥洒汗水干出来、拼出来的结果。在此，我代表市人民政府，向全市人民，向人大代表、政协委员，向各民主党派、工商联、无党派人士和人民团体，向各位老领导、老同志，向驻邯人民解放军、武警官兵、政法干警和消防救援队伍指战员，向中直、省直驻邯各单位，向所有关心支持邯郸发展的同志们、朋友们，致以崇高的敬意和衷心的感谢！

在肯定成绩的同时，我们也清醒认识到，当前经济运行稳中有变、变中有忧，风险和困难明显增多，一些深层次矛盾尚未得到根本解决。产业结构依然偏重，发展质量效益还不高；科技创新能力不强，转型升级步伐不快，新兴产业尚未形成有效支撑；生态环境保护和污染防治形势依然严峻，仍需持续加力攻坚；实体经济发展困难还不少，营商环境还需进一步改善；民生领域仍有不少欠账和短板，优质公共服务供给不足；一些地方和部门作风不实、落实不力，甚至还存在有令不行、有禁不止和消极腐败现象。同时，实际利用外资、规模以上工业增加值等指标没有达到预期目标。对此，我们一定坚持问题导向，采取有力措施，认真加以解决。

各位代表！一年来的实践，使我们更坚定地认识到：必须坚持把习近平新时代中国特色社会主义思想作为最根本的政治引领、最强大的思想武器、最重要的行动指南，武装头脑、指导实践、推动工作。必须坚持贯彻新发展理念，摒弃惯性思维和路径依赖，崇尚创新、注重协调、倡导绿色、厚植开放、推进共享，自觉践行绿水青山就是金山银山的理念，努力实现更高质量、更有效率、更加公平、更可持续的发展。必须坚持深化供给侧结构性改革，坚决去、主动调、加快转，努力实现从高排放、高耗能、高污染、低收益的老“三高一低”，向高技术、高精尖、高效益、低排放的新“三高一低”转变，以高质量发展引领邯郸转型升级、跨越提升。必须坚持走改革开放创新之路，坚决破除不合时宜的思想观念和体制机制障碍，进一步解放思想，创新竞进，激发经济社会发展的强大活力。必须坚持以人民为中心的发展思想，千方百计解决好群众最关心最直接最现实的问题，使群众在教育、就业、社保、医疗、居住、环境等方面有更多实实在在的获得感、幸福感和安全感。

各位代表！上下同欲者胜，同舟共济者赢。在这个千帆竞发、百舸争流的时代，只要我们紧紧依靠全市人民，以功成不必在我的精神境界和功成必定有我的责任担当，逢山开路，遇河架桥，争做新时代邯郸改革发展奋进路上的奔跑者和追梦人，就一定能战胜各种风险挑战，就一定能把邯郸各项事业推向前进！

二、2019年目标任务和主要工作

今年是新中国成立70周年，是全面建成小康社会、实现第一个百年奋斗目标的关键之年。当今世界面临着百年未有之大变局，我国发展仍处于并将长期处于重要战略机遇期，加快经济结构调整、提升科技创新能力、深化改革开放、加快绿色发展、参与全球治理体系变革，丰富了我国战略机遇期的新内涵。习近平总书记亲自谋划、亲自推动的京津冀协同发展、雄安新区规划建设、冬奥会筹办在河北同期实施，使我省重大机遇叠加交汇，形成了强大的发展动力和独特的发展优势。当前，邯郸正处在转型升级、爬坡过坎的关键时期，危和机同生共存，困难中蕴藏着机遇，挑战中孕育着希望。历经40年改革开放，邯郸拥有雄厚的工业基础和广阔的市场空间，特别是连续7年实施项目“三集中”，不仅为经济持续稳中向好蓄能增势，更锤炼了干部担当实干的作风，激发了全市上下创新创业的激情，我们完全可以在新一轮竞争中化危为机、转危为安，变短板为潜力，化挑战为机遇，实现换道超车、后发先至。只要我们坚定不移沿着习近平总书记指引的方向前进，紧紧抓住历史性窗口期和战略性机遇期，就一定能走出一条创新发展、绿色发展、高质量发展的光明大道，开创邯郸更加美好的未来。

今年政府工作的总体要求是：坚持以习近平新时代中国特色社会主义思想为指导，全面贯彻党的十九大和十九届二中、三中全会精神，深入落实习近平总书记对河北工作重要指示批示和中央经济工作会议部署，按照省委九届八次全会和市委九届五次全会要求，统筹推进“五位一体”总体布局，协调推进“四个全面”战略布局，坚持稳中求进、底定超干，坚持新发展理念，坚持推动高质量发展，坚持以供给侧结构性改革为主线，坚持深化市场化改革、扩大高水平开放，加快建设现代化经济体系，继续打好三大攻坚战，统筹推进稳增长、促改革、调结构、惠民生、防风险工作，进一步稳就业、稳金融、稳外贸、稳外资、稳投资、稳预期，提振市场信心，增强人民群众获得感、幸福感、安全感，保持经济持续健康发展和社会大局稳定，为全面建成小康社会收官打下决定性基础，以优异成绩庆祝中华人民共和国成立70周年。

经济社会发展的主要预期目标是：全市生产总值增长6.5%左右，一般公共预算收入增长6%左右，规模以上工业增加值增长4.5%左右，固定资产投资增长6%以上，社会消费品零售总额增长9.5%左右，实际利用外资、进出口总值完成省定目标；城镇登记失业率控制在4.5%以内；城镇、农村居民人均可支配收入均增长7%左右；居民消费价格涨幅控制在3%左右；森林覆盖率达到33.7%；PM2.5平均浓度下降7%，单位生产总值能耗和化学需氧量、二氧化硫、氨氮、氮氧化物等主要污染物排放量完成省定目标。

今年改革发展稳定任务繁重，必须按照省委九届八次全会和市委九届五次全会要求，坚持政治引领、稳中竞进、创新驱动、改革开放、保障民生、高质量发展，着力在十个方面攻坚突破：

（一）着力稳定经济增长，在提高质量效益上攻坚突破。推动供给结构与需求结构有效匹配、投资消费出口“三驾马车”协同发力，构建多元支撑的增长动力格局。以高质量投资优化供给结构。强化高端高质项目引领，开展“重点项目质量年”活动，突出抓好格力智能装备基础件产业基地、中建材碲化镉发电玻璃等40个省重点项目，实施300个市重点项目，完成年度投资800亿元以上。充分发挥政府投资撬动作用，加大生态环保、公路、水利、社会民生、新型城镇化等10个领域补短板力度，实施121个市本级投资项目，完成年度投资150亿元。继续坚持“三集中”机制和“四色”管理法，围绕“532”产业，加大项目谋划、引进、建设力度，更加注重投资完成率和开工率、实际纳税额、亩均投资和产出、能耗、水耗等关键指标核算，全面提高项目质量效益，提高全要素生产率。以高质量供给扩大消费需求。适应消费增速加快、比重提升新趋势，加快文化、旅游、医疗、康养等服务业发展。促进文化与旅游融合发展，加强与中文在线、华强集团、华侨城等企业合作，新增30家骨干文旅企业，确保中华成语文化博览园等项目投入运营。深度融入京津冀旅游协同发展，挖掘特色资源，打造精品线路，加速旅游营销和品牌一体化建设，抓好大运河文化带邯郸段建设，支持武安办好第四届市旅发大会、广平举办开春旅游活动，加快创建省全域旅游示范区，全市旅游总收入增长20%以上，努力打造“河北文旅强市”“中国文旅名城”。培育康养产业，促进医、养、旅、居、文、体等相关产业融合发展。深入推进“增品种、提品质、创品牌”行动，让群众吃得放心、穿得称心、用得舒心。落实个人所得税专项附加扣除政策，增加居民收入，增强消费能力。以高质量出口带动产业升级。实施“开拓国际市场年”活动，推动出口市场多元化，新增外贸龙头企业20家以上、中小外贸企业200家以上，争取20家以上邯商外贸企业回乡发展。发展贸易新业态新模式，深化武安保税物流中心与河港集团合作，支持青岛保税港区邯郸（鸡泽）功能区跨境电商海关监管作业场所运营、永年标准件市场申报国家市场采购贸易方式试点，推进邯郸中欧班列常态化运营。

（二）着力加快转型升级，在产业结构调整上攻坚突破。坚持以供给侧结构性改革为主线，落实“巩固、增强、提升、畅通”八字方针，依托雄厚的制造业基础，着力强链、补链、延链，通过工业设计优化一批、技术改造提升一批、科技创新转化一批、产业延伸集聚一批，加快构建现代化经济体系。以市场化法治化手段，大力破除无效供给。落实好重点行业和钢铁行业去产能三年工作方案，严格执行环保、质量、技术、能耗、水耗、安全等标准和规划、土地、社保、税收四个“杠杠”，关小促大、保优压劣，推动总量性去产能向结构性去产能转变，年内压减退出煤炭产能75万吨、焦炭产能120万吨，完成省定钢铁去产能年度任务。大力推进钢铁企业减量重组、退城进园，启动邯钢老区退城整合提升，加快华信、太行、冀南三大钢铁项目建设，提升钢铁产业集中度和竞争力。积极稳妥处置“僵尸企业”，建立健全优胜劣汰机制，通过兼并重组一批、债务重组一批、破产重组一批、破产清算一批等方式，加快推动市场出清，释放大量沉淀资源，加快腾笼换鸟。以提升产业链水平为方向，推动产业向中高端迈进。靠“强链”促进传统产业高质量发展。深入开展“千企转型”行动，实施技改项目335个以上、完成投资700亿元以上，技改投资增长6%以上，力争2020年前规模以上工业企业全部实施一轮技术改造。靠“补链”壮大战略性新兴产业。实施高技术产业化示范、创新百强培育等6大工程，大力发展新材料、新能源汽车、大数据物联网和信息技术、生物医药健康等战略性新兴产业，抓好718所肥乡特种气体二期、瑞通通信纳米半导体、晨光大健康天然植物提取产业基地等一批大项目，抓好省级战略性新兴产业示范基地建设，构筑产业体系新支柱，高新技术产业增加值增长12%以上。加快推进中船重工（邯郸）军民融合产业示范区建设，全市军民融合型企业达到70家左右。传承发展中医药产业，推进科研、种植、加工、医疗、康养全产业链协同发展，建设中医药强市。加大个转企、小升规力度，新增入统工业企业100家以上。靠“延链”推动先进制造业与现代服务业深度融合。加快工业设计发展，建设区域一流市工业设计创新中心。实施“企业上云”计划，培育建设钢铁、装备、食品、建材、白色家电等行业级工业互联网平台，加速制造业数字化、网络化、智能化转型，抓好“互联网+先进制造业”试点示范项目培育，全面提升邯郸“智造”水平。加快发展金融、会展、电子商务、智慧物流等现代服务业，谋划建设空港物流园区，推进陆港、空港“双港”联动，加快冀南快递产业园、唯品会中部城市群总部、中铁加仑邯郸LNG综合物流园等项目建设。促进“互联网+”和“+互联网”相协同、线上和线下相结合、虚拟和现实相交互，打造新业态，创造新经济。服务业增加值增长10%左右。以实施制造强市、质量强市和标准化战略为引领，增强产业核心竞争力。牢固树立和践行大质量、大标准观，推进标准、质量、品牌、信誉联动提升，主导或参与制定国际、国家标准5项以上。深入开展质量管理提升工程，组织实施工业品、农产品、服务产品、文化旅游产品“四品创牌”行动，新增中国驰名商标3件、河北知名品牌55项、省优质产品30项，使“邯郸制造”更具竞争优势，“邯郸设计”更具品牌价值，“邯郸服务”更具质量信誉。

（三）着力改善生态环境，在污染防治上攻坚突破。认真落实习近平生态文明思想，坚守阵地、巩固成果，持续攻坚、扩大战果，扎实打好每场战役，在治理污染、修复生态中营造良好人居环境。坚决打赢蓝天保卫战。调整优化产业结构、能源结构、运输结构、用地结构，加快形成绿色发展方式。坚持以气定改、以电定改，完成清洁取暖改造55万户左右。深入实施工业企业排放达标计划，完成18家钢铁、21家焦化企业超低排放改造，全市8台35蒸吨/小时及以上燃煤锅炉完成超低排放改造，全面提升工业企业无组织排放治理标准，精准制定治理措施。推进重点企业烟气“消白”治理，力争符合条件的全部完成整治。推广清洁生产、绿色制造，实施创建“白名单”企业计划，今年力争新增50家“白名单”企业。提高“六个三”整治标准，严格主城区道路扬尘“双五双十”和“以克论净”，全面落实建筑施工扬尘控制“六个百分百”和颗粒物（PM10）在线监测、远程视频监控“两个百分百”。加强机动车监管和尾气治理，完成机动车辆尾气排放检测平台和21个固定式尾气遥感监测点建设，构建“天、地、人、车”一体化监管模式。继续提高铁路货运能力和比例，有铁路专用线的15家重点企业铁路运输平均占比达到70%以上。建立“散乱污”企业常态排查机制，实现动态清零。整合优化生态环境大数据平台，建立健全监控与执法信息共享机制，有效应对重污染天气，精准开展重点行业差别化管控，科学实施差异化错峰生产，严禁“一刀切”，进一步提高治污的科学性和精准性。坚决打赢碧水攻坚战。深入实施水污染防治行动计划，严格落实河（湖）长制，对滏阳河、渚河、洺河等7条河流实行“一河一策”，持续推进达标治理，对全市18条重点河渠实施跨界断面水质监测全覆盖。加快市区和县城、开发区污水处理厂及配套管网建设，完善19个重点镇污水集中处理设施，稳步推进建制镇污水集中处理设施建设。全面落实城市黑臭水体治理三年工作方案，确保城市建成区80%以上水体稳定达标。对18个县级集中式饮用水水源保护区进行专项整治，全市地表水国省考断面优良比例达到20%以上。坚决打赢净土守护战。扎实推进土壤污染状况详查，开展工业用地和农用地治理修复，强化污染废弃物全流程管控，抓好土壤污染修复，严格耕地保护，减少高耗水农作物种植面积和化肥农药使用量，下大力解决农业面源污染、白色污染问题。持续开展整治侵占生态空间等专项行动，依法从严惩处环境违法行为。同时，深化生态环境大排查大整治，全面推进生态保护修复，抓好露天矿山深度整治，完成37处关闭取缔和19处责任主体灭失矿山迹地修复绿化。推进地下水超采综合治理，形成压采能力5000万立方米，把恢复地下水位纳入考核内容。治理水土流失面积150平方公里，上半年关停自备井134眼。大规模开展国土绿化行动，高质量完成营造林60万亩以上，加快创建国家森林城市。不折不扣抓好中央和省环保督察“回头看”反馈意见整改落实，举一反三，建章立制，严厉打击环境违法行为。倡导简约适度、绿色低碳的生活方式，全年推广3000辆新能源标准车，大力发展绿色建筑、装配式建筑，广泛开展节约型机关、绿色家庭、绿色学校、绿色社区等创建活动，营造崇尚生态文明的社会氛围。

（四）着力释放发展活力，在深化改革开放上攻坚突破。以改革破解难题，以开放聚集资源，按下“快进键”，跑好“接力赛”，为全市经济发展注入新的活力。深化重点领域改革。深入推进“放管服”改革，建立市场准入负面清单动态调整机制，实施企业开办提速、证照分离等专项行动，项目审批时间再压减一半，继续推进“多证合一”登记制度改革，70%以上政务服务事项实现“一窗”分类受理，市、县两级各100个高频事项实现“最多跑一次”。全面推进“互联网+政务服务”建设，优化环保、消防、税务、市场监管等执法方式，实行企业申请、受理、核准、发照、公示网上全流程办理，实现审批“不见面”。深化工程建设项目审批制度改革，狠抓“五个一”建设，进一步优化审批流程，强化监管服务。深化国有企业改革，全面启动六大系统72家企业改制破产和职工安置，对市级经营性国有资产集中统一监管，加快城发、建投、交投、水投、市政、文体旅等六大国有企业集团改革，积极推进混合所有制改革。深化预算绩效改革，做好消费税等税制改革准备。深化投融资体制改革，规范有序进行PPP合作。扩大全方位对外开放。抓实招商引资“一号工程”，系统完善考核激励机制，围绕产业链招商，市县两级建立一条产业链、一个工作方案、一支招商团队的工作机制，突出对接央企、国际国内企业500强、民营企业500强、上市公司，积极承接京津和长三角、珠三角产业转移，提高招商精准性和实效性。聘请境外招商代理3家、国内招商代理5家，积极组织参加各类招商活动。深度融入“一带一路”建设，支持武安新峰、文安公司在埃及、马来西亚建设钢铁项目。提升开发区能级水平。实施开发区提档升级行动，深化管理体制和运行机制改革，高标准编制开发区主导产业发展规划，省级以上开发区主导产业经营收入占比达到70%以上。支持邯郸经济技术开发区、冀南新区建设京津冀产业转移承接平台，鼓励有条件的县（市、区）以“园中园”“微中心”等模式承接京津产业转移，积极组织我市开发区与京津开发区、总部企业、科研院所等合作共建产业园区。发展壮大民营经济。认真贯彻习近平总书记在民营企业座谈会上的重要讲话精神，全面落实各项惠企强企政策，深入开展减税降费专项行动，对小微企业和科技型初创企业实施普惠性税收减免，降低社保缴费率，降低用能和物流成本，清理和规范各种附加收费，力争今年降低实体经济成本30亿元。实施民营企业“十百千”提升工程，培育更多的“专精特新”民营企业。搭建政银企对接平台，拓宽民营企业融资渠道，市县建立民营企业纾困基金。打破各种“卷帘门”“玻璃门”“旋转门”，在市场准入、审批许可、招投标等方面一视同仁，给民营企业发展创造充足市场空间。构建亲清新型政商关系，给予企业家更多关爱和荣誉，依法保护企业家人身和财产安全，每年发布民营企业百强、民营企业制造业百强、民营企业服务业百强、高新技术企业名单。全力打造市场化法治化营商环境。以企业需求为导向，进一步营造稳定公平可预期的营商环境。持续深化“双创双服”活动，继续实行重点企业和重点项目包联制度，严格规范涉企行政行为，当好“店小二”，做好企业“服务员”。严厉打击假冒伪劣等违法行为，对各类损害营商环境的现象零容忍、严惩处，在全社会形成亲商、安商、富商的浓厚氛围，树立办事便捷、服务周到、安定有序、重诺守信的邯郸形象。

（五）着力增添发展动能，在强化创新驱动上攻坚突破。大力推动科技创新与经济社会发展深度融合，积极创建国家级创新型城市试点，筑牢现代化经济体系战略支撑。培育壮大创新主体。继续实施科技倍增计划，新增高新技术企业100家、科技型中小企业1200家、省级以上创新平台13家。加大财政投入力度，引导企业加大研发投入，全社会研发经费支出占生产总值比重提高到1.25%。用足用好创新要素。抢抓京津冀协同发展战略机遇，深化与中科院、北京大学、清华大学、中国农大、北科院等高校院所合作，北大邯郸创新研究院完成10个科研项目成果转化，北科院邯郸分院设立科技孵化器、创新中心、工业设计中心和科普中心。提升区域创新能力。围绕先进装备制造、新材料等特色优势产业，发挥行业骨干企业主导作用，构建一批产业技术创新战略联盟，支持冀南新区争创国家级高新技术产业开发区。实施县域创新驱动发展跃升计划，支持曲周县争创国家级创新型试点县，新增省级创新型试点县（区）2家以上，加快农业科技园区和可持续发展试验区建设，打造区域创新示范引领高地。全年专利申请量达到5000件，万人发明专利拥有量达到1.75件。激发创业创新活力。继续实施孵化器和众创空间倍增计划，年内新增省级以上科技企业孵化器2家、星创天地3家、众创空间5家，扩大知识产权质押融资，用好双创专项金融债券和孵化专项债券。引进培养创新人才。实施人才强市工程，出台关于支持人才创新创业的若干意见，实行更有含金量、更具吸引力的人才政策，积极引进一批尖端科研团队、领军人才和高层次、高技能人才，推动人才公寓建设，营造优越的创新创业环境。

（六）着力推进乡村振兴，在农业农村现代化上攻坚突破。坚持农业农村优先发展，以实施乡村振兴战略为总抓手，对标全面建成小康社会必须完成的硬任务，切实抓好“三农”工作。推进农业全面升级。深入实施农业供给侧结构性改革、现代农业园区、都市现代农业建设等系列三年行动计划，促进一二三产业融合发展。大力调整粮经饲结构，大力推进“一减四增”，大力发展科技农业、绿色农业、品牌农业、质量农业，构建农业全产业链条，培育提升高端设施蔬菜、中药材、果品、畜牧等特色产业，新建10个市级以上现代农业产业园区，粮食产量稳定在100亿斤以上，打造京津冀都市圈绿色优质农产品供应基地。实施农产品加工业提升行动，抓好100个农产品加工项目，全市农业产业化经营率达到70%。改善农村人居环境。强力推进农村人居环境三年整治行动，重点抓好垃圾污水处理、厕所革命、村容村貌提升、“四好农村路”建设，发动群众搞清洁、搞绿化、搞建设、搞管护，形成持续推进机制，行政村主街道硬化率、村域生活垃圾收集处理率均达到90%以上，建设一批宜居宜业宜游的美丽乡村。深化农村综合改革。完善农村承包地“三权分置”制度，稳慎抓好宅基地“三权分置”改革，确保农村集体产权制度改革国家级整市试点工作通过验收。鼓励引导工商资本参与乡村振兴，培育家庭农场、农民合作社等新型经营主体，全市土地流转率达到35%以上。深入推进强乡富村。全面启动“消零消薄”工程，今年村集体收入空白村实现清零。培育新型职业农民，培养一批农业职业经理人、乡村工匠、文化能人。完善乡村治理机制，建设平安乡村，倡导移风易俗，弘扬文明家风、良好乡风、淳朴民风。

（七）着力提升城市能级，在加快新型城镇化上攻坚突破。按照习近平总书记“一尊重、五统筹”的要求，积极融入以首都为核心的世界级城市群，加快建设以海绵城市、绿色城市、智慧城市、人文城市等为核心内涵的新型城市，全力打造京津冀联动中原的区域中心城市，常住人口城镇化率提高1.2个百分点左右。完善城市规划。坚持全域规划，统筹“五级两规一导则”，推进“多规合一”，对标雄安新区规划建设理念，启动城市国土空间规划编制，科学安排生产、生活、生态三大空间，提高规划的科学性、前瞻性、系统性。提速城市建设。加强5G基站、人工智能、物联网等新型基础设施建设，系统完善城市供热、供气、供电、供水、排涝、污水和垃圾处理等市政设施，全市建设垃圾焚烧发电项目5个，推进主城区大外环、邯郸机场三期、高铁存车线等项目建设，加快城市轨道交通、京石邯城际铁路、聊邯长客专等项目前期工作，提升重大基础设施保障水平。全面推进东区建设，完成年度投资50亿元以上，突出抓好新兴财富广场等中央商务区项目建设，开工建设市第一医院新院区、东部排水防涝区综合治理项目，加快推进综合路网、地下综合管廊建设，新开河竣工通水，建设高品质城市新区。加快老城区改造提升，推进“城市双修”，串城街历史文化街区整治项目竣工投用，实施16个城中村改造项目，完成364个老旧小区改造，开工改造棚户区3000套、基本建成1000套。继续用好“金角银边”闲置土地，建成20个街头游园、停车设施和城市驿站，打通6条断头路、小区占用城市道路。强化城市精细化管理。进一步理顺城市管理体制，运用大数据、互联网、手机APP等新技术提档升级数字化城管系统，常态化创建文明城市，积极创建国家卫生城市、国家生态园林城市，精心筹办省第四届园博会和第三届城市设计大赛，建设康湖生态文明示范区。繁荣发展城市经济。培育壮大楼宇经济、总部经济，积极引进金融、科技、结算中心等高端企业，加快提升城市首位度和核心竞争力。促进县城扩容提质。扎实开展县城建设三年突破行动，以县城新区和教育园区建设为带动，扩大县城规模，完善基础设施，促进产城教融合发展，提高县城和小城镇建设水平。大力发展县域经济。落实激励县域经济发展的财政政策，因地制宜打造特色产业，加强配套协作，推动县域经济特色化、特色经济产业化、产业经济集群化。深化巩固“大棚房”整治成果，长效推进房地产市场平稳健康发展。

（八）着力营造安全环境，在防范化解重大风险上攻坚突破。安不忘危，兴不忘忧。我们一定坚持底线思维，增强忧患意识，严格落实责任，牢牢把握风险防控主动权。坚决防控金融风险。全面开展重点领域再排查再整治，严厉打击非法集资、恶意逃废债行为，加大处置化解不良贷款力度。建立健全市县两级地方金融监管体系，对接“河北金融云”平台，推进金融风险排查、化解、防控、监管、考评常态化，坚决守住不发生系统性、区域性金融风险底线。支持金融业持续健康发展，引导银行向企业发放中长期贷款，落实降低小微企业融资担保费率奖补机制，鼓励企业通过发行企业债、中期票据等方式融资，力争引进3家金融机构、新增融资300亿元，推进2家企业上市取得实质性突破，提高金融服务实体经济能力。坚决防控债务风险。按照“坚定、可控、有序、适度”原则，深入开展地方政府隐性债务统计监测，建立化解台账，严格执行地方政府债务限额管理制度，打开“前门”规范举债融资，积极稳妥化解债务存量，堵住“后门”杜绝盲目举债，切实遏制隐性债务增量。坚决防控安全风险。坚持生命至上、安全第一，建立统一的应急管理信息系统，构建综合高效的应急处置协调机制。建立健全风险分级管控和隐患排查治理双重预防工作机制，推行风险清单、隐患清单管理。持续开展安全隐患大排查大整改活动，深化矿山、冶金、危险化学品、道路交通、建筑施工、城乡燃气、消防、人员密集场所等行业领域安全生产专项整治，严防各类事故发生。加大食品药品监管力度，开展药品、保健品非法营销专项整治，确保“舌尖上的安全”。坚决防控社会风险。认真解决欠薪、房地产遗留等信访问题，切实维护群众合法权益。推进扫黑除恶专项斗争向纵深开展，推动重点案件快侦快办，严格落实“一案三查”，彻底打掉“关系网”和“保护伞”，在打防并举、标本兼治上下功夫。以“雪亮工程”为载体，打造立体化、信息化治安防控体系。强化网络安全和信息化建设，制止和打击利用网络传播违法违规信息的行为，营造清朗的网络空间。

（九）着力夯实小康基础，在精准脱贫上攻坚突破。把脱贫攻坚作为重大政治任务和第一民生工程，一鼓作气、尽锐出战，重点解决好实现“两不愁、三保障”面临的突出问题，确保3.3万贫困人口脱贫，44个贫困村出列，实现所有贫困县、贫困村脱贫出列。提高精准识别质量。开展基层基础工作规范提升行动，强化扶贫信息的精准和共享，做到“脱贫即出、返贫即入”。严格落实非贫困县财政扶贫资金每年人均不少于4000元标准，推进非贫困县、非贫困村贫困人口按计划稳定脱贫。持续促进稳定增收。扎实推进产业扶贫“十百千”示范工程，实施特色种养、电商等六大产业扶贫工程，推广建设“扶贫微工厂”，加快实现产业扶贫项目贫困户全覆盖，统筹推进就业、科技、生态扶贫。兜住贫困群众底线。确保教育、健康、住房等保障性扶贫政策落实，开展贫困地区健康扶贫攻坚行动，进一步提升基本医疗保险、大病保险、医疗救助三重医疗保障和贫困户家庭医生签约服务水平。打好易地扶贫搬迁、农村危房改造和“空心村”治理组合拳，确保搬得出、稳得住、能致富。完善防贫长效机制。探索“群体式参保、基金式管理、社会化经办、阳光化操作”防贫运行机制，深入开展“千企帮千村”行动和结对帮扶，坚持脱贫不脱政策，强化对“摘帽”县和脱贫户后续扶持，加大对非持续稳定脱贫户、非建档立卡低收入户等“边缘户”的扶持保障力度，确保全面小康路上不让一人掉队。强化脱贫监督管理。深化扶贫领域腐败和作风问题专项治理，实施扶贫对象、扶贫政策、扶贫资金、扶贫项目“四公开”，严肃查处违纪违法行为。

（十）着力增进人民福祉，在补齐民生短板上攻坚突破。坚持尽力而为、量力而行，完善制度、守住底线，进一步加大民生投入，着力实施20项民心工程和10项民生实事，让改革发展成果更多更公平地惠及全市人民。稳定提升就业水平。重点抓好高校毕业生、农民工、退役军人、下岗转岗职工等群体就业，新增城镇就业11.26万人、失业人员再就业3.24万人、困难人员实现再就业1.2万人，确保零就业家庭动态清零。大规模开展职业技能培训，全年培训各类技能人才3.8万人次。优先发展教育事业。贯彻全国和全省教育大会精神，推进学前教育普及普惠优质安全发展、义务教育优质均衡发展、高中教育特色多样发展、职业教育产教融合发展、高等教育高质量内涵式发展，成立市产学研委员会，加快推进邯郸职教城建设，筹建邯郸科技职业学院、冀南技师学院，河北工程大学新校区投入使用，谋划组建邯郸城市大学，全市新建、改扩建乡村小规模学校和乡镇寄宿制学校校舍14万平方米、幼儿园50所。深入实施主城区中小学幼儿园建设三年行动计划，开工建设17个项目。全面加强社会保障。深入推进全民参保计划，深化企业养老保险制度改革。完善低保、特困精准认定和指导标准动态调整机制，最低指导标准增长10%左右，主城区居家养老服务中心覆盖率达到80%左右。关心关爱特困人员、残疾人、优抚对象等群体，为6448名贫困残疾人提供基本康复服务，为4200名贫困残疾人免费适配基本型辅助器具，为1979名贫困残疾人提供托养服务补贴。切实强化医疗保障。完善统一的城乡居民基本医疗保险和大病保险制度，推进跨省异地就医直接结算。建立完善医药品集中招标采购新机制，推动基本医疗保险支付方式改革，把更多救命、救急的好药纳入医保，全过程监控医疗行为，从根本上降低药品和医药耗材价格，切实解决老百姓“看病贵”问题。加强社保基金、医保基金、企业年金、职业年金监管。加快建设健康邯郸。贯彻实施“健康中国人”行动计划，推进医疗、医保、医药、医价“四医联动”改革，组建3所医疗集团，推进30家城市社区卫生服务中心与大医院组建医联体、30家乡镇卫生院和村卫生室一体化管理试点。建设“生命大健康”智慧服务平台，宣传倡导“治未病”理念，积极创建全国医养结合试点城市、全国分级诊疗试点城市。深入开展“互联网+医疗健康”便民惠民活动，建设更多更好的“健康小屋”，使群众在家门口就能享受良好的医疗服务。大力发展文体事业。积极培育和践行社会主义核心价值观，完善现代公共文化服务体系，积极推进市美术馆、市文史馆、市方志馆建设，市博物馆新馆、市群艺馆年内投用，实施中华优秀传统文化传承发展工程，开展优秀传统文化进乡村、进社区、进校园活动，打造太极文化、成语文化、赵文化品牌，组织各级各类演出1000场以上。精心举办好第三届中国吹歌节、第十三届市运动会等大型文体活动，创建省全民健身公共服务体系示范区，新建城乡体育健身设施300处以上。加强国防教育和国防后备力量建设，强化退役军人服务保障，巩固军政军民团结的好局面。统筹做好民族宗教、新闻出版、广播电视、邮政通信、外事侨务、史志档案、人民防空、防灾减灾、援藏援疆、妇女儿童等各项工作。

各位代表！群众利益无小事，民生工作大于天。我们一定说到做到，带着感情和责任办好民心工程、民生实事，坚决兑现向全市人民的承诺！

三、全面加强政府自身建设

实现今年的目标任务，使命在肩，责无旁贷。我们一定以习近平新时代中国特色社会主义思想为指导，时刻把发展的重任扛在肩上，把人民的利益放在心头，以许党许国的担当、只争朝夕的精神、攻坚克难的勇气，争当推动高质量发展的“急先锋”，坚决做到信念过硬、政治过硬、责任过硬、能力过硬、作风过硬，努力创造无愧于党、无愧于人民、无愧于时代的崭新业绩。

（一）旗帜鲜明讲政治，坚定正确政治方向。把政治建设摆在政府建设的首要位置，坚持用习近平新时代中国特色社会主义思想指引政府工作，强化“四个意识”，坚定“四个自信”，坚决维护习近平总书记党中央核心、全党的核心地位，坚决维护党中央权威和集中统一领导，始终在政治立场、政治方向、政治原则、政治道路上同党中央保持高度一致。严守政治纪律和政治规矩，坚决落实党中央国务院、省委省政府和市委决策部署，确保政令畅通、令行禁止。深入开展“不忘初心、牢记使命”主题教育，铸牢永远听党话跟党走的坚定信仰，锤炼对党绝对忠诚的政治品格。

（二）依法行政重规范，切实维护公平正义。严格遵守宪法和法律，做社会主义法治的忠实崇尚者、自觉遵守者、坚定捍卫者。加强重大决策合法性审查，严格落实法律顾问制度，加强行政机关负责人出庭应诉和行政复议工作。健全行政决策责任追究制度，加快构建决策科学、执行坚决、监督有力的权力运行机制。主动接受市人大及其常委会法律监督和工作监督、市政协民主监督，认真办理人大代表建议和政协提案，广泛听取民主党派、工商联、无党派人士、人民团体和各界人士的意见建议，扎实推进政务公开和政府信息公开,让行政权力始终在阳光下运行。

（三）履职尽责勇担当，全面提高工作效能。强化责任担当，一级带着一级干，一级做给一级看，一级为一级解难，一级对一级负责，勇挑最重担子，敢啃最硬骨头，抓铁有痕、踏石留印，以担当带动担当，以作为促进作为。强化工作落实，坚持“四个干”“六个点”工作法，迎着困难上、奔着问题去，苦干实干、久久为功，把各项工作做到位、做极致、做精彩。强化效率提升，只争朝夕、雷厉风行，夙兴夜寐、激情工作，以奔跑的状态真抓实干，真正顶起自己该顶的那片天。强化严明奖惩，着力整治“为官不为”，健全激励和容错纠错机制，旗帜鲜明为敢于担当、踏实做事、不谋私利的干部撑腰鼓劲。

（四）不忘初心解民忧，自觉践行为民宗旨。民之所想，政之所向。坚持问政问计于民，把人民拥护不拥护、赞成不赞成、高兴不高兴作为谋划工作、制定政策、推动发展的依据，顺应民心、尊重民意，让人民共享改革发展成果。始终保持对人民的赤子之心，大兴调查研究之风，推动工作重心下移，察民情、接地气，面对面、心贴心，真正做到以人民忧乐为忧乐、以人民甘苦为甘苦。走好网上群众路线，及时回应群众通过《人民网地方领导留言板》、市政府网站、961890热线等平台反映的问题，建立健全发布、解读、回应“三位一体”政务公开体系，切实回应重大民生关切。

（五）从严治政不懈怠，始终保持廉洁本色。高标准推进政府系统党风廉政建设和反腐败斗争，坚持无禁区、全覆盖、零容忍，坚持重遏制、强高压、长震慑，巩固发展反腐败斗争压倒性胜利，一体推进不敢腐、不能腐、不想腐，取得全面从严治党更大战略性成果。深化集中整治形式主义、官僚主义成果，严肃查处空泛表态、应景造势、敷衍塞责、出工不出力等问题。严格落实中央八项规定及其实施细则精神，带头遵守《准则》和《条例》，深化中央和省委巡视整改落实工作，在常和长、严和实、深和细上下功夫。强化权力运行监督，加大审计监督力度，严厉整治查处侵害群众利益的不正之风和腐败问题。全体公务员要知敬畏、存戒惧、守底线，习惯在接受监督和约束的环境中工作生活。

各位代表！伟大梦想不是等得来、喊得来的，而是拼出来、干出来的！让我们更加紧密地团结在以习近平同志为核心的党中央周围，在省委、省政府和中共邯郸市委的坚强领导下，齐心协力，埋头苦干，锐意进取，奋力开启改革开放新征程，以新时代全面建设富强邯郸、美丽邯郸的新业绩，庆祝新中国成立70周年！
